# Role of Anti-ganglioside Antibodies in the Diagnosis of Guillain-Barré Syndrome as an Alternate Investigation

**DOI:** 10.7759/cureus.4625

**Published:** 2019-05-09

**Authors:** Muhammad Luqman Saeed, Behzad Kaleem Baloch, Syed Nayer Mahmud, Muhammad Tariq Khan, Muhammad Shoaib Safdar Qureshi, Zahid Siddique Shad, Syed Waqar Hussain, Kamran Munawar, Aayesha Qadeer, Azmat Abdullah

**Affiliations:** 1 Internal Medicine, Shifa International Hospital, Islamabad, PAK; 2 Nephrology, Shifa International Hospital, Islamabad, PAK; 3 Internal Medicine, Khan Research Laboratories Hospital, Islamabad, PAK

**Keywords:** anti-gangliosides antibodies, gbs diagnosis, alternate investigation

## Abstract

Objective

The goal of the study was to see if anti-ganglioside antibodies have a role in the diagnosis of Guillain-Barré syndrome (GBS).

Study design

Between May 2016 to October 2017, we conducted a prospective pilot study of 15 patients with a clinical diagnosis of GBS with equivocal cerebrospinal fluid (CSF) analysis and/or nerve conduction studies (NCS) .

Materials and methods

All adult patients (age >18 years) whose clinical diagnosis was GBS but diagnostic tests (either NCS or CSF analysis or both) were not suggestive of GBS were included in the study and were tested for anti-gangliosides antibodies. Data was entered in SPSS, version 21.0 (IBM, Armonk, New York) and analyzed.

Results

Of the 15 patients fulfilling the inclusion criteria, 60% had a normal CSF analysis while 40% had normal NCS. The percentages of different GBS variants observed in sampled patients were acute inflammatory demyelinating polyradiculopathy (AIDP) 40%, acute motor axonal neuropathy (AMAN) 40%, acute motor and sensory axonal neuropathy (AMSAN) 13.3%, and Miller Fisher syndrome 6.7%. However, the anti-ganglioside antibodies were negative in all patients.

Conclusion

Anti-gangliosides antibodies cannot be used as an alternative diagnostic investigation in GBS patients as our study failed to show positive results in different GBS variants.

## Introduction

Guillain-Barré syndrome (GBS) is a group of neuropathic conditions which is characterized by progressively increasing weakness and diminished or absent reflexes [1-2]. The annual incidence of GBS in the United States is around 1.65 to 1.79 per 100,000 [3].

The underlying mechanism involved in the pathogenesis of GBS is the formation of anti-gangliosides antibodies which are formed most commonly after Campylobacter jejuni (C. jejuni) infection due to the mechanism of molecular mimicry. The cell wall of C. jejuni expresses lipo-oligosaccharides whose structure is similar to gangliosides of the nerves. Different types of anti-gangliosides antibodies can be formed on the basis of cell wall structure and can involve different parts of the neuron [3].

GBS can be classified into acute inflammatory demyelinating poly-radiculoneuropathy (AIDP), acute motor and sensory axonal neuropathy (AMSAN), and acute motor axonal neuropathy (AMAN) on the basis of different sites involved by antibodies [4].

GBS can be diagnosed on the basis of clinical features, cerebrospinal fluid (CSF) testing, and nerve conduction studies(NCS) [3]. Protein levels in CSF may be normal in early GBS, but they are elevated in 90% of patients by the end of the second week of symptoms [5]. The normal CSF white blood cell count helps differentiate GBS from other infectious, inflammatory, and malignant diseases. However, GBS may produce an elevated CSF white blood cell count in patients who are serologically positive for human immunodeficiency virus (HIV) [6]. Electro-diagnostic study results may be normal in up to 13% of patients soon after symptom onset, but rarely remain normal on sequential testing over the initial weeks of symptoms [7]. Anti-ganglioside antibodies are reported as positive in 36% of patients with GBS and become positive early in the disease process. Isotypes were, immunoglobulin G (IgG) (62%), IgG + IgM (26%) and IgM (12%) [8-9].

Anti-gangliosides are of six different types corresponding to the six immuno-clinical variants of GBS:

1) Antibodies to ganglioside GM1 (anti-GM1) and GD1b IgG and IgG > IgM in the acute motor axonal neuropathy after C. jejuni infection;

2) anti-GD1a IgG in severe motor axonal GBS after C. jejuni infection;

3) anti-GQ1b IgG in Miller Fisher syndrome;

4) anti- GT1b ganglioside and polysialogangliosides IgG in cranial nerve variants;

5) anti-GD1b IgG in pure ataxic sensory GBS;

6) anti-GM2 IgM in severe GBS with antecedent cytomegalovirus (CMV) infection.

These autoantibodies can differentiate between suspected motor peripheral neuropathies and motor neuron diseases (sensitivity 73%, specificity 83%, positive predictive value 60%, negative predictive value 91%) [10]. Specifically, antibodies to ganglioside GM1 are present in 14%-50% of patients with GBS, and are more common in cases with severe axonal degeneration associated with any subtype.

The role of different types of anti-gangliosides antibodies in the diagnosis is not clear but it can be used in cases where difficulty arises in the differentiating GBS from other diseases, or when the common diagnostic tests come out to be negative or inconclusive before starting costly treatments; delaying treatment can increase morbidity. In addition, CSF examination can be contraindicated in conditions like bleeding diathesis and local infections. In this study, we checked anti-gangliosides antibodies in those patients suspected as having GBS but the diagnostic tests were not suggestive of GBS.

## Materials and methods

This pilot study and was carried out at the departments of neurology, medical ICU and nephrology of Shifa International Hospital, Islamabad, Pakistan. The study was approved by the ethical committee and was funded by the Shifa Clinical Research Center. The study was carried out over a period of one and a half years. Inclusion and exclusion criteria were made.

All patients with a clinical diagnosis of GBS or one of its variants (based on preceding history of gastrointestinal or respiratory systems, neuromuscular weakness with or without cranial nerves involvement, and appropriate examination findings) were included. Nerve conduction studies and CSF analysis of all patients was done. Those patients who had either equivocal NCS or CSF analysis or both, were included in the study. Due to the limited availability of funds, patients who had unequivocal NCS results and CSF analysis were excluded from the study.

Preceding events before the diagnosis of GBS like respiratory tract infection, diarrhea, and history of vaccination were recorded along with the duration of disease symptoms. The patient’s anti-gangliosides antibodies titers were sent as an anti-ganglioside profile which included all six types of aforementioned anti-ganglioside antibodies on the first day of admission and the standard treatment and levels were correlated to the residual disability after treatment. The test was performed by enzyme-linked immunosorbent assay (ELISA). The functional ability of patients during the course of the disease and after treatment was scored using the disability score on treatment of GBS (0, normal; 1, able to run; 2, able to walk 5 m unaided; 3, able to walk with aids; 4, not able to walk, bedbound or chair bound; 5, intubated, on artificial ventilation). Cranial nerve involvement was scored as none (0), present (1) and requiring intervention (2; tube feeding). Correlation between anti-gangliosides antibodies titer and level of weakness was noted. A total of 15 patients were enrolled in the study. All collected data was entered in SPSS, version 21.0 (IBM, Armonk, New York). Continuous variables like age, duration of symptoms, anti-gangliosides antibodies titer, level of weakness score are presented as mean +- standard deviation (SD), and categorical variables like sex are presented as proportions (%). Correlations of the anti-gangliosides antibodies as an alternate diagnostic evaluation in GBS and between weakness score and anti-gangliosides titers pre and post-treatment was also established by using a chi-square test. A p-value of <0.05 is considered significant.

## Results

The study duration was of one and a half years. Fifteen patients who met the inclusion criteria were enrolled in the study; serological testing for anti-ganglioside antibodies was carried for all of them. Overall, seven patients were males (46.7%) and eight (53.3%) were females and age ranged from 18 to 55 years with a mean of 35.2 and SD of +/- 1.22. Days of symptoms onset prior to presentation was different for different patients but the minimum day of presentation was one and maximum of four days with a mean of 2.33 +/- 0.975 SD. Preceding event before GBS onset was diarrhea in 73.3% of cases and respiratory tract infection in 26.6% of cases. The percentages of different GBS variants observed in sampled patients were AIDP 40%, AMAN 40%, AMSAN 13.3% and Miller Fisher syndrome 6.7%. Regarding diagnostic evaluation, CSF findings were normal in nine out of 15 patients with a percentage of 60% and NCS findings were normal in six out of 15 patients with a percentage of 40%. Detailed results are given in the tables below (Tables 1-7). The anti-gangliosides antibodies were negative in all the sampled population with the percentage of 0%. Similarly, the level of muscle weakness and cranial nerves abnormalities were noted pretreatment but not after treatment because of negative antibodies test pre-procedure and correlation could not be drawn between the degree of weakness and its associated antibodies titer.

**Table 1:** Age in years

Age	Frequency	Percent
18	1	6.7
19	1	6.7
20	1	6.7
22	1	6.7
28	1	6.7
30	1	6.7
33	2	13.3
40	1	6.7
42	1	6.7
45	1	6.7
46	1	6.7
47	1	6.7
50	1	6.7
55	1	6.7

**Table 2:** Days of symptoms onset prior to presentation

No. of days	Frequency	Percent
1	3	20.0
2	6	40.0
3	4	26.7
4	2	13.3

**Table 3:** Source of infection before onset

	Frequency	Percent
Diarrhea	11	73.3
Respiratory tract infection	4	26.7

**Table 4:** GBS variant GBS: Guillain-Barré syndrome; AIDP: acute inflammatory demyelinating polyradiculopathy; AMAN: acute motor axonal neuropathy; AMSAN: acute motor and sensory axonal neuropathy.

	Frequency	Percent
AIDP	6	40.0
AMAN	6	40.0
AMSAN	2	13.3
Miller Fischer	1	6.7

**Table 5:** Nerve conduction study (NCS) findings

	Frequency	Percent
Demyelinating neuropathy	4	26.7
Motor axonal neuropathy	3	20.0
Motor and sensory axonal neuropathy	2	13.3
Normal ncs	6	40.0

**Table 6:** Cerebrospinal fluid (CSF) findings

	Frequency	Percent
Normal protein	9	60.0
Raised protein	6	40.0

**Table 7:** Descriptive statistics

	N	Minimum	Maximum	Mean
Level of muscular weakness	15	2.00	5.00	3.4000
Cranial nerve weakness	15	.00	2.00	.2000

## Discussion

We investigated the role of anti-gangliosides antibodies as an alternate diagnostic test in GBS. We checked these antibodies in GBS patients in whom either one or both of the diagnostic tests like CSF or NCS came out as negative. GBS can be diagnosed on the basis of clinical features, CSF testing, and NCSs [3]. Protein levels in CSF may be normal in early GBS, but they are elevated in 90% of the patients by the end of the second week of symptoms [5]. The normal CSF white blood cell count helps differentiate GBS from other infectious, inflammatory, and malignant diseases. Electro-diagnostic study results may be normal in up to 13% of the patients soon after symptom onset, but rarely remain normal on sequential testing over the initial weeks of symptoms [7].

Anti-ganglioside antibodies are reported as positive in 36% of patients with GBS and became positive early in the disease process [11]. Gangliosides are chemical compounds located on neural tissue of axon, and antibodies when formed, binds against these chemical compounds. Different types of antibodies are present in different GBS variants [12-13] like anti-GM1 and GD1b in AMAN, anti-GD1a IgG in severe motor axonal GBS, anti-GQ1b IgG in Miller Fisher syndrome, anti- GT1b ganglioside and polysialogangliosides IgG in cranial nerve variants, anti-GD1b IgG in pure ataxic sensory GBS and anti-GM2 IgM in severe GBS with antecedent CMV infection [9]. In contrast to the literature, our study did not show any positive test result for anti-gangliosides antibodies in all of the sampled 15 patients.

Different types of precedent infections occur before the GBS onset like C. jejuni diarrhea, Haemophilus influenza infection, CMV infection with intestinal C. jejuni infection being the most common amongst all with the reported percentage of around 30% in patients with GBS [14]. The underlying mechanism involved in the pathogenesis of GBS is molecular mimicry between lipo-oligosaccharides of C. jejuni and different gangliosides of neural tissue leading to cross-reaction of antibodies against C. jejuni with gangliosides. Pathogenetic pathways other than mentioned above are the formation of antibodies against Schwann cells, myelin sheaths and glycolipids other than those are the targets of C. jejuni located in axons 2 and 28. In our study, 11 out of 15 showed precedent infection with diarrhea before the development of GBS but surprisingly anti-gangliosides antibodies were negative in all those 11 patients. Although serology for C. jejuni was not checked in these patients, it can be assumed from the study that either a different pathogenetic mechanism was involved in GBS development or some organism other than C. jejuni might be involved in those with precedent diarrhea. In addition to molecular mimicry, there are other ways in which GBS can develop. These include the development of antibodies against sodium ion channels on the neuronal axons thereby blocking the conduction of neuronal impulses. The treatment in such cases includes the administration of 7S-immunoglobulins [15-16].

## Conclusions

Anti-gangliosides antibodies cannot be used as an alternative diagnostic investigation in GBS patients as our study failed to show positive results in different GBS variants. Similarly, the study also shows that a different pathogenic mechanism might be involved in GBS development in this part of the world but the results cannot be generalized as the sample size was small and research on a larger scale might be required to prove this.
